# Taxifolin Enhances Andrographolide-Induced Mitotic Arrest and Apoptosis in Human Prostate Cancer Cells via Spindle Assembly Checkpoint Activation

**DOI:** 10.1371/journal.pone.0054577

**Published:** 2013-01-28

**Authors:** Zhong Rong Zhang, Mazen Al Zaharna, Matthew Man-Kin Wong, Sung-Kay Chiu, Hon-Yeung Cheung

**Affiliations:** Research Group for Bioactive Products, Department of Biology and Chemistry, City University of Hong Kong, Hong Kong SAR, China; Institut de Génomique Fonctionnelle de Lyon, France

## Abstract

Andrographolide (Andro) suppresses proliferation and triggers apoptosis in many types of cancer cells. Taxifolin (Taxi) has been proposed to prevent cancer development similar to other dietary flavonoids. In the present study, the cytotoxic and apoptotic effects of the addition of Andro alone and Andro and Taxi together on human prostate carcinoma DU145 cells were assessed. Andro inhibited prostate cancer cell proliferation by mitotic arrest and activation of the intrinsic apoptotic pathway. Although the effect of Taxi alone on DU145 cell proliferation was not significant, the combined use of Taxi with Andro significantly potentiated the anti-proliferative effect of increased mitotic arrest and apoptosis by enhancing the cleavage of poly(ADP-ribose) polymerase, and caspases-7 and -9. Andro together with Taxi enhanced microtubule polymerization *in vitro*, and they induced the formation of twisted and elongated spindles in the cancer cells, thus leading to mitotic arrest. In addition, we showed that depletion of MAD2, a component in the spindle assembly checkpoint (SAC), alleviated the mitotic block induced by the two compounds, suggesting that they trigger mitotic arrest by SAC activation. This study suggests that the anti-cancer activity of Andro can be significantly enhanced in combination with Taxi by disrupting microtubule dynamics and activating the SAC.

## Introduction

Andrographolide (Andro) is a diterpenoid lactone isolated from the functional herb *Andrographis paniculata*, which has been used as a folk medicine to treat upper respiratory infections, sore throats and many other infectious diseases. This compound has been suggested to have great potential for cancer treatment because it shows anti-cancer activities in both direct and indirect ways [1]–[5]. Andro can directly inhibit tumor cell growth through the induction of cell cycle arrest, apoptosis and differentiation, and it can indirectly suppress cancer development through immunostimulating, anti-inflammatory, anti-angiogenic and chemoprotective properties [4]. Andro may modify the p50 subunit of NF-kB in an antagonistic manner, and this mechanism may be the cause of its multiple activities [6]. Its drug activity is dependent on its concentration because it can suppress apoptosis in many cell types at certain concentrations [7], [8], [9], but it can also induce apoptosis at high concentrations [10], [11], [12]. Many studies have demonstrated that Andro effectively induces cell cycle arrest in several types of cancer cells at the G0/G1 stage [10], [13], [14] and in human hepatoma HepG2 cells at the G2/M phase [15]. Our recent study shows that the effects of Andro are dependent on the cell type. For example, it arrests the cell cycle at G2/M in several hepatocellular cancer cell lines, but it leads to apoptosis in HeLa cells [16]. According to previous studies, Andro activates the extrinsic death receptor pathway and induces apoptotic cell death in different types of cancer cells. In most cell types, effector caspase activation also requires amplification of the signal via a mitochondria-dependent apoptotic pathway [11], [17]. Pro-apoptotic Bcl-2 family members (Bid and Bax) are believed to play key roles as mediators in the relay of the cell death signal stimulated by Andro [11], [12].

The role of dietary flavonoids in cancer prevention has also been widely discussed. Compelling data from laboratory studies and human clinical trials indicate that flavonoids play an important role in cancer prevention and treatment [18]–[20]. A substantial number of studies have demonstrated that flavonoids are able to potentiate the anti-tumor activity of other agents on multiple types of cancer [21]–[24]. Taxifolin (Taxi), also known as dihydroquercetin, has a strong anti-oxidant effect and possesses activities similar to quercetin, which enhances apoptosis induced by a variety of anti-cancer agents in both cell and animal models [25]–[28]. However, the combined use of Taxi with other anti-cancer agents has not been reported.

Although Andro has been reported to exert an anti-proliferative effect on many types of cancer cells [29], detailed molecular studies of its effect on androgen-refractory prostate cancer cells are still lacking. We hypothesized that multi-target treatment is a promising method to make use of the anti-cancer activities of naturally occurring compounds. The present study was undertaken to determine the individual and combined effects of Andro and Taxi on cell cycle arrest and apoptosis in androgen-independent prostate cancer DU145 cells. The apoptotic effects of Andro on the cells were significantly enhanced when used in combination with Taxi by enhancing the apoptotic pathway and inducing the activation of the spindle assembly checkpoint (SAC).

## Materials and Methods

### Cell culture and chemicals

Human androgen-independent prostate carcinoma cells DU145, immortalized human prostate cells PNT2, and normal human foreskin fibroblasts Hs27 were obtained from the American Type Culture Collection (MD, USA). These cell lines were routinely maintained in DMEM supplemented with 10% fetal bovine serum (FBS) (Invitrogen, CA, USA), 0.37% sodium bicarbonate, 100 units/ml penicillin and 100 µg/ml streptomycin at 37°C in a humidified 5% CO_2_ air incubator. Purified Andro (97%) was obtained from Indofine Chemical Company (NJ, USA). Taxi that was 85% pure was purchased from the Sigma-Aldrich Corporation (MO, USA) and a 98% pure Taxi preparation was purchased from BioBioPha (Yunnan, P.R.C.). Both preparations had the same effect on cell viability when given alone or in combination with Andro. Both chemicals were first dissolved in DMSO (less than 0.1%) and then diluted with complete growth medium to the final desired concentration before treatment. Samples treated with DMSO alone served as vehicle controls. All other reagents were obtained from Sigma-Aldrich unless otherwise indicated.

### MTT cell proliferation/viability assay

Cell proliferation and viability were assessed by 3-(4, 5-dimethylthiazol-2-yl)-2, 5-diphenyl-tetrazolium bromide (MTT) (Invitrogen, CA, USA) assay. DU145 cells, PNT2, and Hs27 cells were seeded at 5000 and 1000 cells/well, respectively, into a 96-well plate. The cells were incubated for 24 h, and then exposed to various concentrations of Andro, Taxi or a mixture of the two for a predetermined period of time. At the end of the treatment, the drug-containing medium was removed and 0.5 mg/ml MTT dissolved in the same medium was added to each well. The plate was further incubated for an additional 3 h. After removing the supernatant at the end of incubation, 100 µl DMSO was added to each well to dissolve the formazan crystals that had formed. The absorbance at 570 nm was measured with a multi-well scanning spectrophotometer (Model 550, Bio-Rad, USA). Cell proliferation was expressed as percentage of control by comparing the number of live cells in the treatment group to the number in the vehicle group.

### Observation of cellular and nuclear morphology

Cells were seeded on sterilized coverslips with an approximately 30% confluence, followed by 24 h of incubation and chemical treatment. The cells were washed briefly with PBS and stained with 5 µg/ml of Hoechst 33342 (Invitrogen, CA, USA) in non-FBS DMEM for 20 min at room temperature. Morphological changes in the cells were examined using phase contrast and fluorescent microscopes (Mikron Instruments, NY, USA).

### Cell cycle analysis using flow cytometry

Control and treated cells were harvested and fixed in 70% ice-cold ethanol overnight at 4°C. After removing ethanol and washing with PBS, the fixed cells were resuspended in a DNA staining solution containing 50 µg/ml propidium iodide (PI) and 100 µg/ml RNase A in PBS at a density of about 1×10^6^ cells/ml. They were subsequently incubated for another 30 min at 37°C in subdued light. The stained cell suspension was analyzed with a flow cytometer (Becton Dickinson, CA, USA). The DNA content of 10,000 cells per sample was used to analyze the cell cycle using DNA histograms. The DNA content in the cell-cycle of the analyzed cells was calculated by MODFIT 3.0 software (Verity Software House, ME, USA).

### Quantification of mitotic index by flow cytometry

The control and treated cells were collected and fixed in 4% formaldehyde for 10 min at 37°C. The cells were then permeabilized by slowly adding ice-cold methanol to a final concentration of 90% and incubated on ice for 30 min. Approximately 1×10^6^ cells were resuspended in 100 µl incubation buffer (0.5% BSA in PBS) for 30 min at room temperature. The cells were then incubated with phosphohistone H3 (Ser10) antibody (Cell Signaling Technology, Beverly, MA) for 1 h and then with an FITC-conjugated secondary antibody (Santa Cruz Biotechnology, CA, USA) for another 30 min in the dark at room temperature. Immunostained cells were resuspended in 0.5 ml of propidium iodide (5 µg/ml) in PBS and kept in the dark prior to analysis with a flow cytometer within 24 h. The signal from 10,000 cells was measured to generate a scatter plot.

### Quantification of apoptotic rate by flow cytometry

Apoptosis was assessed using the Annexin V-FITC Apoptosis Detection Kit (Sigma-Aldrich) with the procedure provided by the manufacturer. Briefly, cells on the culture dishes were collected and washed, and approximately 5×10^5^ cells were resuspended in 500 µl of 1× Binding Buffer (10 mM HEPE/NaOH, pH 7.5, 0.14 M NaCl and 2.5 mM Cacl_2_). Then, 5 µl of 50 µg/ml AnnexinV FITC Conjugate (in 50 mM Tris-HCl, pH 7.5, 100 mM NaCl) and 10 µl of 100 µg/ml PI Solution (in 10 mM potassium phosphate buffer, pH 7.4, 150 mM NaCl) were added to the cell suspension. After 10 min of incubation in the dark at room temperature, the fluorescence signal from the cells was measured immediately with a flow cytometer.

### Immunofluorescence microscopy

Cells were seeded at 60% confluence on sterilized cover slips and exposed to the chemicals of interest after 24 h incubation. After a predetermined period of time of treatment with the chemicals, cells were fixed with 4% formaldehyde in PBS for 10 min at room temperature, washed with PBS and permeabilized with 0.25% Triton X-100 in PBS for 10 min. The permeabilized cells were subsequently washed and incubated with incubation buffer (3% BSA in PBS) for 30 min at room temperature to block non-specific binding of antibody. β-tubulin antibody (Cell Signaling Technology) was incubated with the cells for 1 h at room temperature, and then FITC-conjugated goat anti-rabbit IgG (Santa Cruz) and the DNA staining dye Hoechst were added for 1 h in subdued light. At the end of the reaction, the cover slips were rinsed with PBS before being mounted on microscope slides with fluorescence mounting medium (Dako, Denmark). The sealed slides were then examined with a Leica SP5 confocal microscope (excitation at 488 nm for FITC and 365 nm for Hoechst).

### Western blot analysis

Total cell lysate was prepared by lysing the cells in RIPA lysis buffer (150 mM NaCl, 0.1% SDS, 0.5% Sodium Deoxycholate, 1% NP-40, and 50 mM Tris-Cl, pH 7.5) supplemented with the Protease Inhibitors Cocktail Set III (Calbiochem, CA, USA) and 1 mM PMSF at a density of 1–2×10^7^ cells/ml buffer. The cell suspension was agitated for 30 min and then centrifuged at 14,000 rpm for 20 min at 4°C. The supernatant was collected as cell extracts. Next, 50 µg proteins from the cell lysates were separated by 12% SDS-polyacrylamide gel electrophoresis (SDS-PAGE) and electrotransferred onto nitrocellulose membranes (Bio-Rad, CA, USA). The membrane was blocked with 3% BSA or non-fat milk in PBS with 0.05% tween-20. This was followed by incubation overnight with a diluted solution of primary antibody against α-tubulin, caspase-3, caspase-7, caspase-9, p-Cdc2 (Tyr15), cyclin A, cyclin B1, cyclin E2, Myt-1, p21, PARP (Cell Signaling), Bcl-2 (Santa Cruz), Bcl-xL (Invitrogen, CA, USA), or Cdc2 (BD) at 4°C. This was followed by incubation at room temperature with HRP-conjugated antibody (anti-mouse IgG or anti-rabbit IgG) for 1 h. Three independent experiments were performed. The blots were visualized by enhanced chemiluminescence (ECL) using the Western Blotting Luminol Reagent (Santa Cruz). Images developed were captured with an LAS-4000 gel documentation system (Fuji Film, Tokyo, Japan).

### *In vitro* tubulin turbidity assay

The influence of the drugs on microtubule polymerization was monitored using CytoDYNAMIX™ Screen 01 kit (Cytoskeleton Inc., CO, USA). Briefly, the drugs at different concentrations were prepared in DMSO at 10× strength in G-PEM buffer, which contains 80 mM PIPES (pH 6.9), 2 mM MgCl_2_, 0.5 mM EGTA, 1 mM GTP and 5% glycerol. DMSO served as a vehicle control. Subsequently, 10 µl of 10× G-PEM buffer was added into each well of a pre-warmed 96-well plate and allowed to incubate for 2–5 min. Tubulin protein (>97% purity) was mixed with G-PEM buffer at a concentration of about 4 mg/ml and then 90 µl of the tubulin solution was added into each well containing 10 µl of the buffer solution. After shaking, the absorbance at 340 nm was measured every minute for 60 min at 37°C.

### RNAi depletion of MAD2

DU145 cells were seeded in 60 mm plates to provide a confluency of 50–70% in 24 h. The cells were transfected with either one of the two Mad2L1 siRNA (Origene, MD, USA) or the control siRNA in the presence of siTran transfection reagent (Origene) in Opti-MEM medium (Invitrogen, CA, USA) for 24 h. The transfected cells were rinsed one time with PBS and incubated with DMEM (Invitrogen, CA, USA) medium for 24 h and were further incubated for another 24 h in medium containing 20 µM Andro and 100 µM Taxi. The cells were then rinsed one time with PBS, trypsinized, and fixed with 70% ethanol and stored at −20°C overnight. The mitotic index cells were then quantified by flow cytometry based on signals from antibody against phospho-histone H3 (at S10) and propidium iodide.

### Statistical analysis

Statistical analysis was conducted using the Origin 7.5 software (Originlab Corporation, MA, USA) and Excel (Microsoft, Redmond, WA, USA). All data were analyzed from 3 or 4 independent experiments. Results were expressed as mean ± s.d. The differences between samples were analyzed using two-sample Student's t-tests. P-values less than 0.05 were considered statistically significant.

## Results

### Andro is much more cytotoxic than Taxi in inhibiting the growth of prostate cancer cells

**Figure 1:**
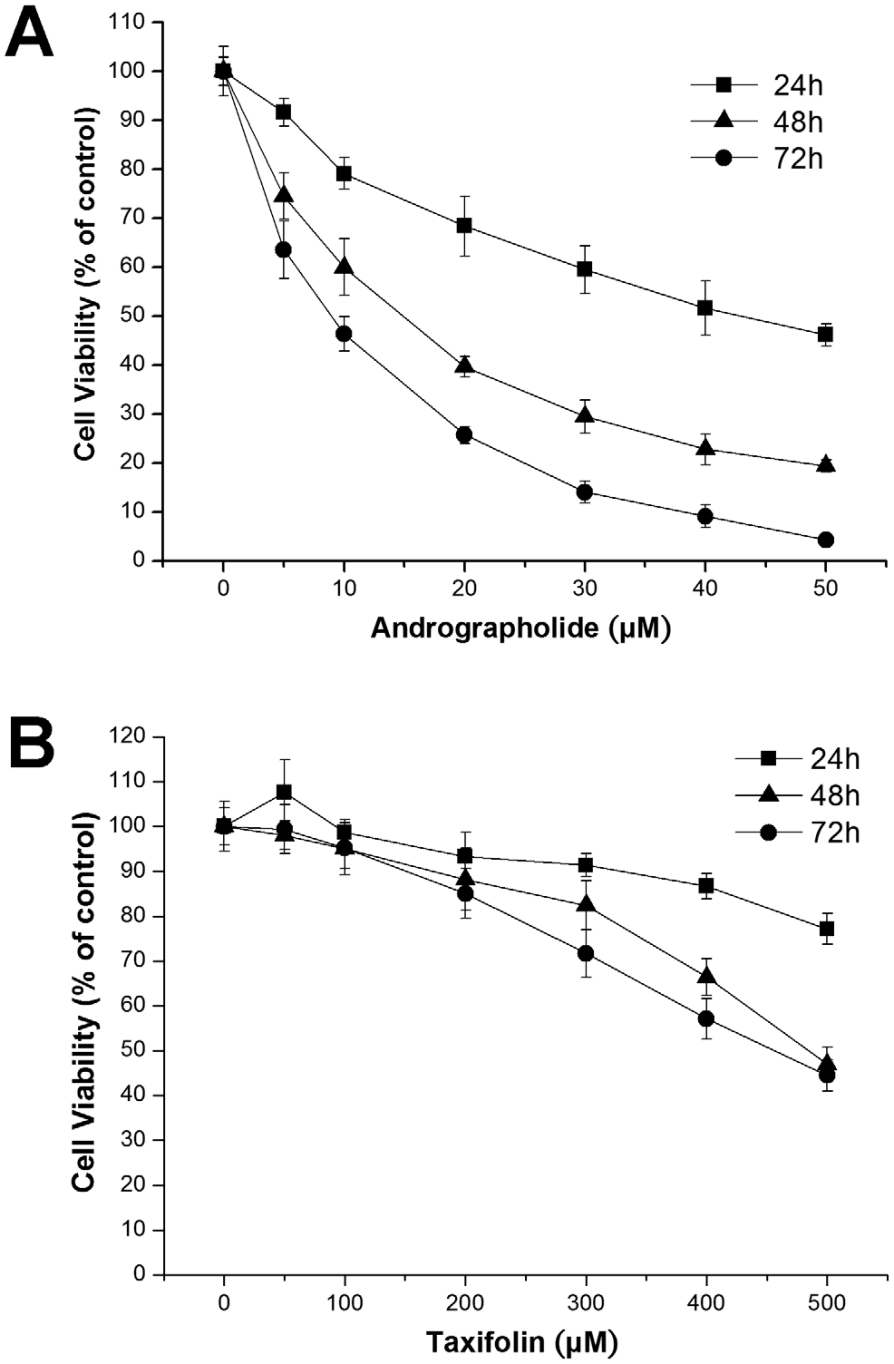
Effect of andrographolide (Andro) and taxifolin (Taxi) on the growth of DU145 cells. The cells were treated with a range of concentrations of (A) Andro and (B) Taxi for the indicated period of time and cell proliferation was monitored by MTT assay. The data from three independent experiments were expressed as percentage of viability compared to the vehicle control (DMSO). Values are mean ± s.d.

Human prostate carcinoma DU145 cells were exposed to 0 to 50 µM Andro for 24, 48 and 72 h, and cell proliferation was assessed using the MTT assay, a common method to measure the metabolic activity of cells to reflect the cell number or proliferation. Andro inhibited the proliferation of DU145 cells in a time- and concentration-dependent manner compared to untreated proliferating cells (Figure 1A). The calculated IC_50_ values of Andro on DU145 cells were 42.76±3.29 (24 h), 13.70±1.45 (48 h) and 8.36±0.77 µM (72 h). The IC_50_ value at 48 h obtained in this study was only slightly higher than the value (12 µM) previously reported by Nanduri *et al.* [29]. At concentrations higher than the IC_50_ value, Andro induced changes in the cellular morphology of treated DU145 cells, which appeared round and shrunken and showed blebbing (Figure S1). These cells also exhibited chromatin condensation and fragmented nuclei (Figure S2).

We also evaluated the cytotoxicity of Taxi on DU145 cells using the MTT assay. The results showed that Taxi is much less cytotoxic to DU145 cells than Andro and that a concentration of 500 µM Taxi was required to inhibit cell growth by 52% after 48 h of exposure (Figure 1B). The result of this study is consistent with a previous report regarding the cytotoxicity of Taxi on mammalian cancer cells [30].

### Andro induces mitotic arrest and apoptosis

**Figure 2:**
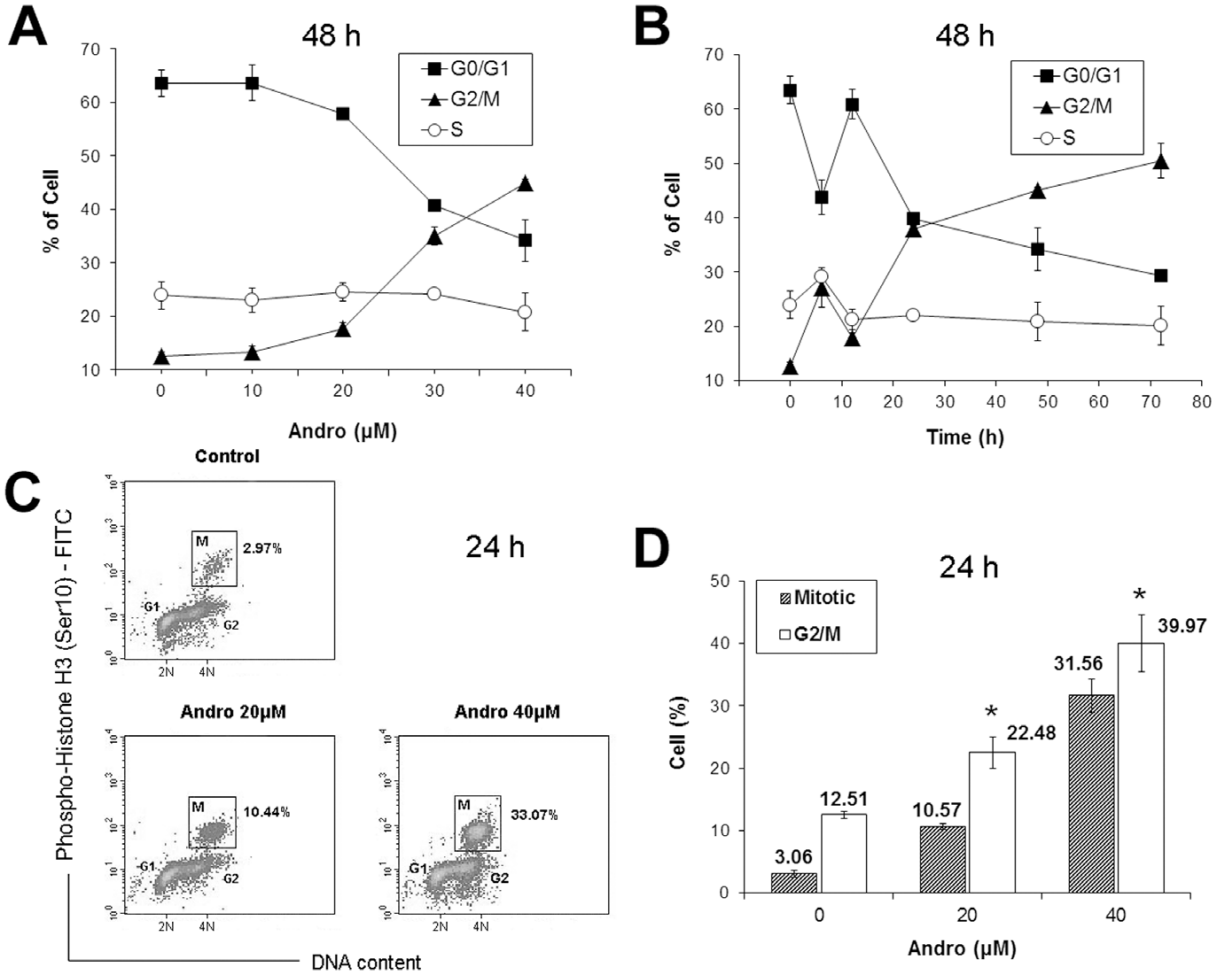
Change in the cell cycle distribution of DU145 cells treated with andrographolide (Andro). (A) Cells were exposed to 0–40 µM of Andro for 48 h; (B) cells were exposed to 40 µM of Andro for 0–72 h. Cellular DNA was stained with propidium iodide (PI) and analyzed by flow cytometry. The percentage of cells in each specific phase of the cell cycle was calculated with the ModFit program and expressed as the mean ± s.d. (n = 3 or 4). (C&D) Mitotic index of DU145 cells treated with Andro. Cells were treated with 0, 20, and 40 µM of Andro for 24 h and were fixed and stained with anti-phospho-histone H3 (Ser10) antibody, FITC-conjugated secondary antibody and PI, and analyzed by flow cytometry. (C) A representative density plot of the cells labeled with phosphohistone H3 against the DNA content in the cells. Mitotic index (M) of the cell population is indicated in the boxed area. (D) Mitotic index and percentage of G2/M cells after treatment with two different concentrations of Andro expressed as mean ± s.d. (n = 4). * indicates significantly different from the control (*p*<0.01).

Cell cycle analysis indicated that treating DU145 cells with Andro at concentrations between 0–40 µM led to an accumulation of cells at G2/M but a decrease in cells at G0/G1 in a concentration-dependent (Figure 2A) and time-dependent manner (Figure 2B). We also determined whether the increase in the G2/M population was due to an increase in mitotic cells at 24 h after Andro treatment. We monitored the mitotic index (the ratio of mitotic cells to the rest of the cell population) by flow cytometry using immunostaining with phospho-histone H3, a mitotic marker, and we observed that Andro induced a significant accumulation of mitotic cells in a dose-dependent manner (Figure 2C). An 11-fold increase in mitotic cells after treatment with 40 µM Andro for 24 h was observed when compared with the vehicle control (the cell population in the rectangles labeled with M in Figures 2C and 2D). A comparison between the mitotic index and the percentage of G2/M cells in the population suggested that the accumulation of G2/M cells was mainly due to mitotic arrest (Figure 2D). This set of experiments demonstrated that after 24 h of treatment with Andro, the cells were primarily in mitotic arrest.

**Figure 3:**
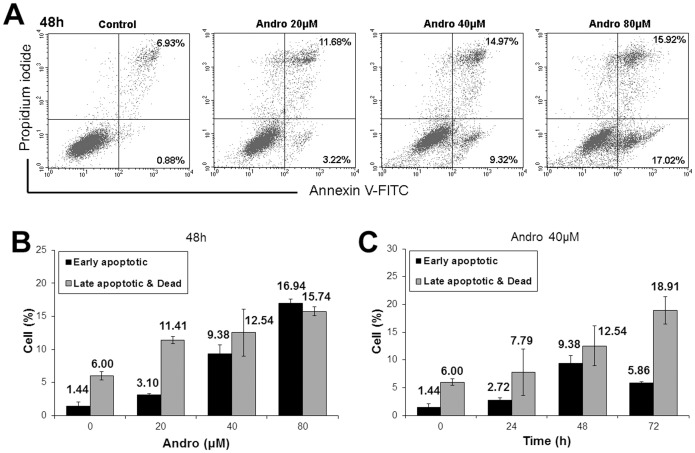
Apoptosis of DU145 cells treated with andrographolide (Andro). (A&B) After treating for 48 h with 0–80 µM of Andro, the cells were collected and doubly stained with Annexin V-FITC antibody and PI before analyzed by flow cytometry. (A) One set of representative dot plots indicates the percentage of early apoptotic cells (Annexin V-FITC^+^/PI^−^, lower right hand panel) and the percentage of late apoptotic and dead cells (Annexin V-FITC^+^/PI^+^, upper right panel). (B) The plot of the percentage of early apoptotic and of late apoptotic and dead cells under different Andro concentrations expressed as mean ± s.d. (n = 4). (C) The cells were exposed to 40 µM of Andro for 0–72 h. The plot of the percent of early apoptotic and of late apoptotic and dead cells against the time of treatment expressed as mean ± s.d. (n = 4).

We also examined conditions that caused Andro to induce apoptosis. Andro-treated cells were stained with Annexin V-FITC and analyzed by flow cytometry. The time-course study of apoptotic rates after treatment with Andro showed that the highest early apoptotic rate (9.38%) occurred at 48 h; however, at 72 h, the early apoptotic rate decreased, and the cell death rate increased (Figure 3C). Moreover, increasing concentrations of Andro affected both early and late apoptosis in a dose-dependent manner (Figures 3A and 3B). We conclude that treatment with Andro inhibited the proliferation of DU145 cells at the G2/M phase and that more of these cells progressed towards apoptosis during longer treatment, with prolonged mitotic arrest leading to apoptosis.

### Effect of Andro on the expression of cell cycle regulators, caspases and Bcl-2 family proteins

**Figure 4:**
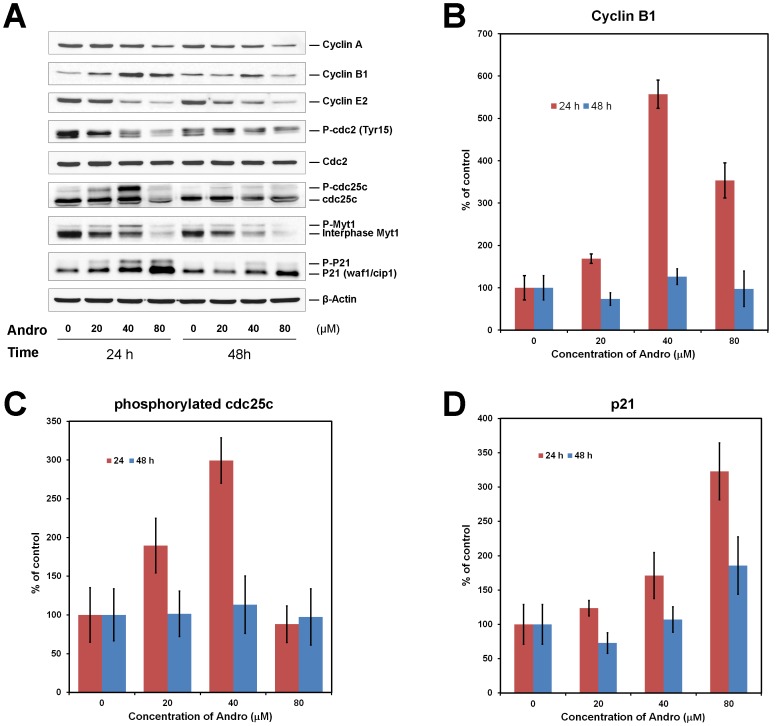
Effect of andrographolide (Andro) on the expression of cell cycle regulators in DU145 cells. (A) The cells were incubated with 0–80 µM of Andro for 24 or 48 h. 40 µg of the cell lysate was resolved by electrophoresis in a 12% polyacrylamide gel, transferred to nitrocellulose membrane, and immunoblotted against the indicated cell-cycle regulator antibodies. Data are the quantification of the western blots of three independent experiments. (B) The change in cyclin B1 level compared with the control (0 µM) from the quantification of the western blots was plotted against the concentration of Andro at 24 and 48 h. (C) The change in the levels of phosphorylated Cdc25c was plotted against the concentration of Andro. (D) The change in the levels of p21 was plotted against the concentration of Andro. Standard deviations of three independent experiments are shown.

To explore the molecular mechanisms of mitotic arrest induced by Andro, the expression levels of several important cell-cycle regulators were analyzed by western blotting. Analysis of the immunoblots showed that 24 h after treatment, a 5.7-fold, 3.2-fold, and 3-fold increase in cyclin B1, p21, and phosphorylated (Tyr15) Cdc25c protein levels, respectively, were observed (Figures 4A–D), whereas the protein levels of cyclin A and cyclin E2 decreased with increasing concentrations of Andro. Clear parallel mobility shifts due to the phosphorylation of Cdc25c, Myt1 and p21 were present at 24 h (Figure 4A). Notably, the expression of cyclin B1 was dependent on the concentration of Andro. For example, its expression was highest at 40 µM but decreased at 80 µM. Similarly, the phosphorylation levels of Cdc25c and Myt1 were the most intense after treatment with 40 µM Andro.

**Figure 5:**
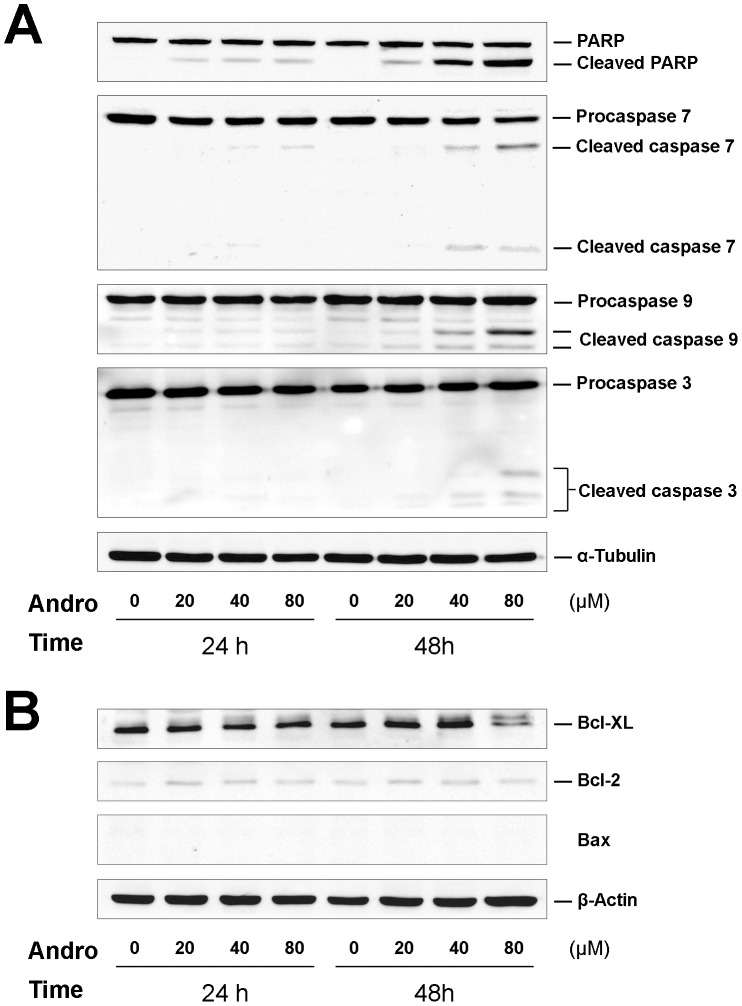
Time course of Andro treatment on caspases and Bcl-2 family proteins expressed in DU145 cells. The cells were incubated with 0–80 µM of Andro for 24 or 48 h, harvested and lysed. 40 µg of the cell lysate was separated in 12% polyacrylamide gel, transferred to a nitrocellulose membrane, and immunoblotted with antibodies against (A) caspase-7, -9, and -3 and substrate PARP; and (B) Bcl-2 family proteins, including Bcl-XL, Bcl-2, and Bax. Data are representative of three independent experiments.

We also examined the expression levels of proteins related to apoptosis, caspases and Bcl-2 family members by immunoblotting with their specific antibodies. Concentration- and time-dependent cleavage of PARP (poly(ADP-ribose) polymerase), caspase 7, 9 and 3 were observed (Figure 5A). The pro-apoptotic protein Bax was not detected either in the control or the treated cells; however, the pro-survival member Bcl-2 was weakly expressed. The expression of Bcl-XL, an anti-apoptotic protein, was clearly detected, but a slower migrating form was observed in treated cells 48 h after exposure to 80 µM Andro (Figure 5B). These data suggest that apoptosis was most significantly induced after 48 h of treatment with 80 µM Andro.

### Taxi potentiates the growth inhibitory effect of Andro

**Figure 6:**
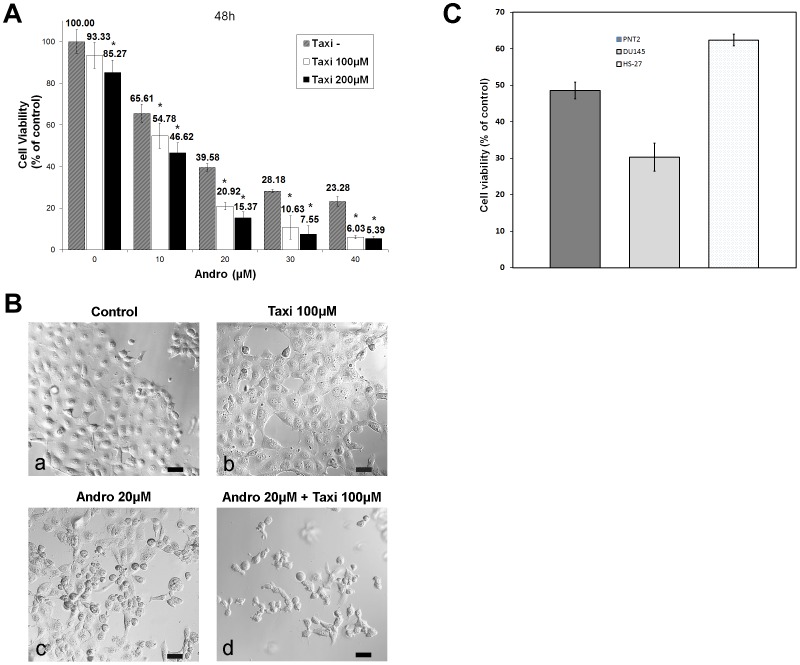
Taxifolin (Taxi) enhances the growth inhibitory effect of Andro on DU145 cells. (A) The cells were treated with 0–40 µM of Andro and 0, 100 or 200 µM of Taxi for 48 h, and cell proliferation was determined by MTT assay. Data from four independent experiments were expressed as percentage of viability compared to the vehicle control (DMSO) with standard deviation (mean ± s.d., n = 4). * indicates significantly difference from the control (*p*<0.01). (B) The cells were treated with 20 µM of Andro for 48 h in the presence or absence of 100 µM of Taxi, and observed under differential interference contrast microscopy. Bar = 50 µm. (C) A comparison of the viability by MTT assay of DU145, PNT2, and Hs-27 cells after the treatment with 20 µM of Andro and 100 µM of Taxi. The experiments were performed in triplicate and the standard deviations are indicated.

We proposed that Taxi may influence the cytotoxic effect of Andro on the cancer cells; therefore, we evaluated the combinatory effect of Andro and Taxi on cell proliferation using the MTT assay. As shown in Figure 6A, when exposed to 100 µM Taxi without any Andro, the relative percentage of proliferating cells was 93.33±6.27% (mean ± s.d., n = 4), which was not significantly lower than the vehicle control (untreated cells). In contrast, a significant decrease in the percentage of proliferating cells (% of control in cell number = 65.6%) was observed in the presence of 10 µM Andro alone, but further addition of 100 µM Taxi resulted in a slightly lower percentage of proliferating cells (54.78%). However, the growth inhibitory effect of 20 µM Andro in combination with 100 µM Taxi (% of control = 20.92±1.89%) was approximately twice the effect of treatment with Taxi alone (% of control = 39.58±1.79%, n = 4), and the difference in cell proliferation occurred when the amount of Taxi was increased in combination with higher Andro concentrations. A similar trend was also observed when 200 µM Taxi was added together with higher concentrations of Andro; however, there was a 14.7% decrease in cell number in the presence of Taxi alone (85.27%), which was significantly lower than that of cells treated with the vehicle control. Therefore, in all subsequent investigations, 100 µM Taxi was used in combination with various concentrations of Andro. The results from the morphological analysis of the treated cells using differential interference contrast microscopy correlated well with the data regarding cell proliferation (Figure 6B). The cells treated with the two compounds showed more shrinkage, lower cell density, a higher number of floating cells and more nuclear condensation and fragmentation (not shown) than the cells treated with Andro alone.

We were interested in testing whether the dual-drug treatment was specific to cancer cells compared to immortalized but non-cancerous cells. The MTT assay was utilized to compare the proliferation of DU145 cells with PNT2 cells, a human prostate cell line immortalized with the SV40 virus, and with Hs-27 cells, a human foreskin fibroblast cell line, treated with 20 µM Andro and 100 µM Taxi. Under the same conditions, only 30.3% of DU145 cells were viable, whereas 48.6% and 62.4% of PNT2 and Hs-27 cells, respectively, were viable (Figure 6C). The result suggests that DU145 cells were more sensitive to the two drugs than the other two immortalized human cell lines.

### Taxi and Andro-induced mitotic arrest led to the accumulation of cyclin B, Cdc25c, and p21

**Figure 7:**
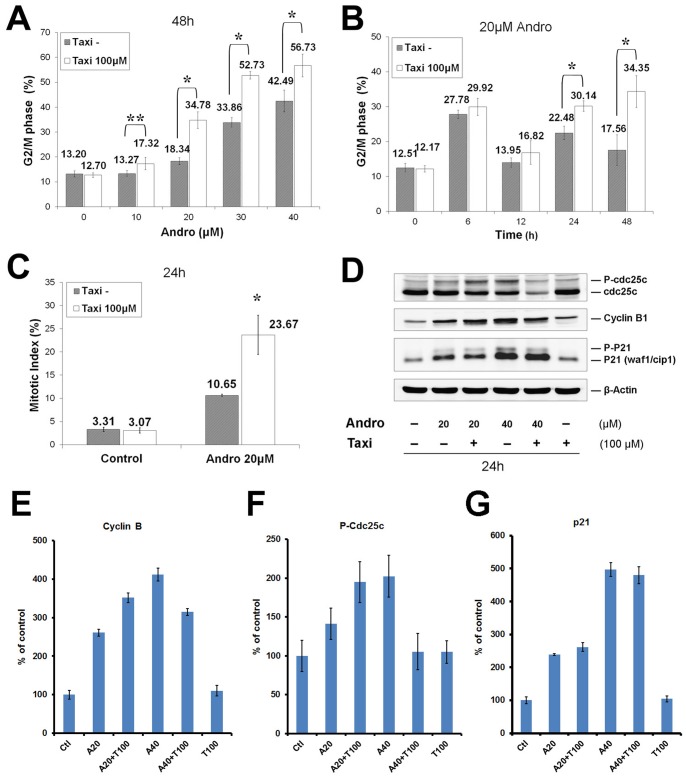
Taxi enhances Andro-induced cell cycle arrest in DU145 cells. The cells were treated with (A) 0–40 µM of Andro for 48 h (B) 20 µM of Andro for 0–48 h in the presence or absence of 100 µM of Taxi. Cellular DNA was stained with PI and analyzed by flow cytometry. The distribution of the cell cycle was calculated with the ModFit program. Mean ± s.d. (n = 4) of percentage of G2/M cell in the presence of Taxi (100 µM) and in the absence of Taxi (Taxi−) were compared. * indicates significant difference between two groups, *p<0.01, **p<0.05. (C) The cells were treated with 20 µM of Andro for 24 h in the presence or absence of 100 µM of Taxi. The treated cells were fixed and doubly stained with anti-phosphohistone H3 (Ser10) antibody and PI, and analyzed with flow cytometry. Mitotic index expressed as mean ± s.d. (n = 3 or 4) in the Taxi− and Taxi 100 µM groups were compared, *p<0.01. (D) Lysates from 40 µg of the cells that had been treated with the indicated Andro and Taxi for 24 h, and then was immunoblotted against antibodies of phosphorylated Cdc25c, cyclin B1, p21, and actin as control. Data are representative of three independent experiments. (E–G) Quantification of the signals from the immunoblots of the cyclin B (E), p-Cdc25c (F), and p21 (G) were plotted under different indicated conditions. The values in the plots are the mean ± s.d. of three independent experiments.

To investigate whether the combined inhibitory effect on the growth of DU145 was due to cell cycle arrest, cell cycle events and mitotic index were assessed in Andro-treated cells with or without Taxi. When Andro was not present, the percentage of cells in G2/M in both groups was not significantly different. However, when the concentration of Andro was increased from 10 µM to 40 µM, the percentage of cells in G2/M in the combined treatment groups was significantly higher than the percentage of cells in the Andro alone group (Figure 7A). Furthermore, significant differences were observed in the culture treated for 24 h with 20 µM Andro in combination with 100 µM Taxi (Figure 7B). The proportion of treated cells in M phase was also measured with the anti-phospho-histone H3 (S10) antibody. The mitotic index of the combined group was approximately twice the level of the cells treated with Andro alone (23.67±4.21% vs. 10.65±0.22%, n = 4), while the mitotic indexes in both control groups did not differ from each other (Figure 7C), suggesting that Taxi alone does not induce an M phase arrest. Immunoblotting was used to analyze the effects of the drugs on the expression levels and modification of Cdc25c, cyclin B1, and p21 proteins (Figure 7D). Increasing the concentration of Andro increased the phosphorylated forms of Cdc25c 2-fold, p21 protein almost 5-fold, and cyclin B1 4.1-fold (Figures 7E–G). After 100 µM Taxi was added to 20 µM Andro, the phosphorylation of Cdc25c and the protein level of cyclin B1 were elevated almost 2-fold and 3.5-fold, respectively. In contrast, the expression level of p21 was not significantly altered in the combined treatment group. However, 40 µM Andro in combination with 100 µM Taxi decreased the cyclin B1 and Cdc25c levels but increased the p21 level by 1.8-fold compared with 20 µM Andro and 100 µM Taxi treatment. These experiments demonstrate that the combined treatment resulted in an accumulation of mitotic arrested cells.

### Taxi promotes Andro-induced apoptosis through further caspase activation

**Figure 8:**
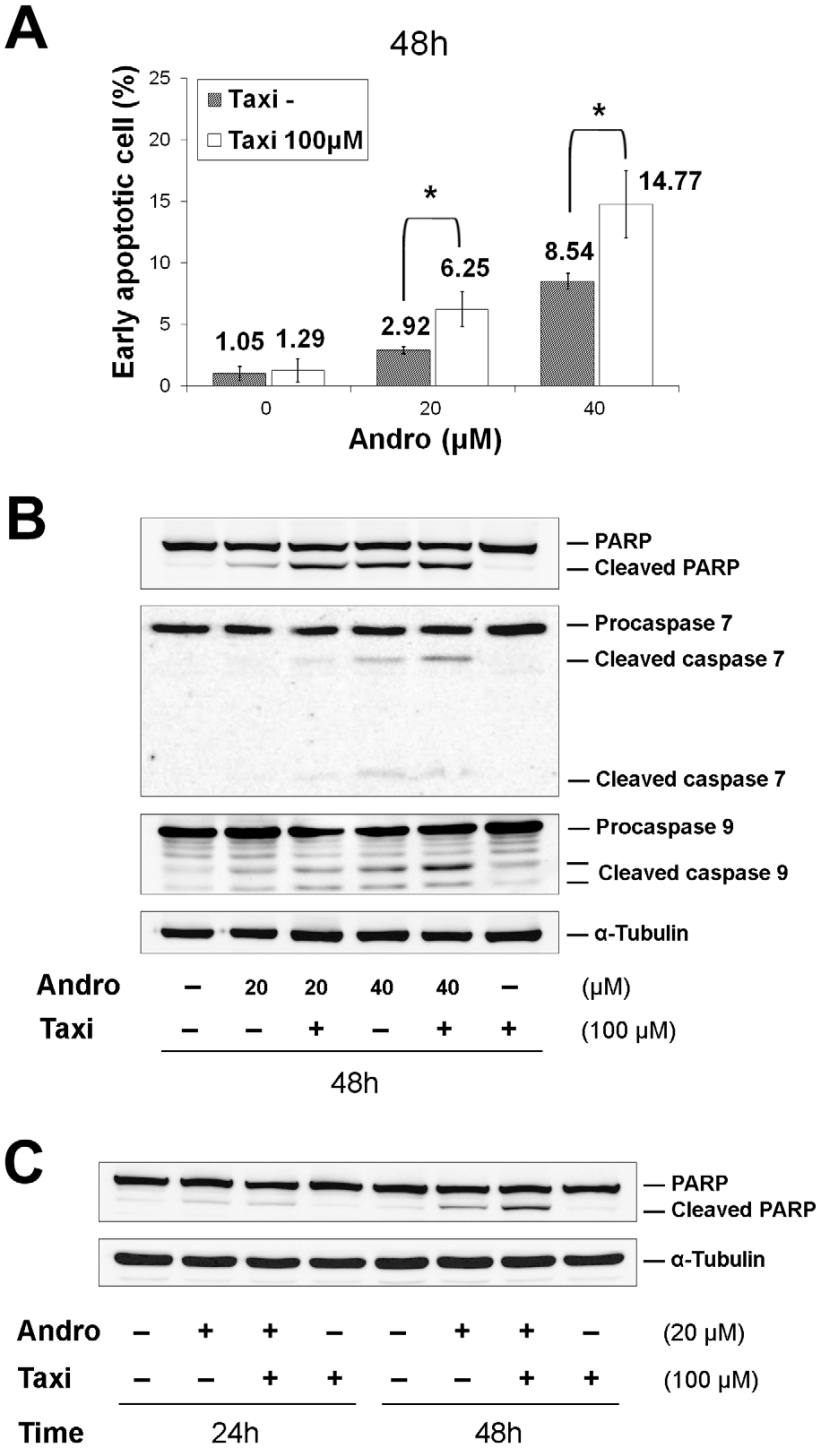
Taxi enhances Andro-induced apoptosis in DU145 cells. The cells were treated with (A&B) 0–40 µM of Andro for 48 h, and (C) 20 µM of Andro for 24 or 48 h in the presence or absence of 100 µM of Taxi. (A) The cells were double stained with Annexin V-FITC antibody and PI and then analyzed with flow cytometry. Mean ± s.d. (n = 4) of percent of early apoptotic cell in the presence of Taxi (100 µM) and in the absence of Taxi (Taxi−) were compared. * indicates a significant difference between the two groups, *p<0.01. (B&C) Western blot analysis of caspase-7, caspase-9, and PARP in DU145 cells. 40 µg of the cell lysates were immunoblotted with antibodies against the caspases and PARP. Data are representatives of three independent experiments.

To quantitatively examine the extent of apoptosis induced by Andro, Taxi and their combination for 48 h, flow cytometry was used to measure the appearance of Annexin V on the cell surface to quantify the progression of the early apoptotic event. The apoptotic rates in the presence or absence of Taxi were concurrently measured. The combination treatment markedly elevated the early apoptotic rate from 2.92±0.29% to 6.25±1.42% when using 20 µM Andro and from 8.54±0.65% to 14.77±2.73% (n = 4) with 40 µM (Figure 8A). In the drug-treated cells, we also measured the cleavage of poly(ADP-ribose) polymerase (PARP), which is another hallmark of early events in apoptosis, as well as procaspase 7 and procaspase 9, which are important proteases in mitochondria-mediated apoptosis. Increasing concentrations of Andro alone induced more cleavage of PARP, procaspase 7 and procaspase 9, and further addition of Taxi led to increased cleavage of these three proteins (Figure 8B). The enhanced cleavage of these three proteins appeared to correspond well with the increased apoptotic rate (Figure 8A). Moreover, longer treatment (48 h) further enhanced PARP cleavage (Figure 8C).

### The combination of Andro and Taxi interferes with microtubule dynamics and activates the spindle assembly checkpoint

**Figure 9:**
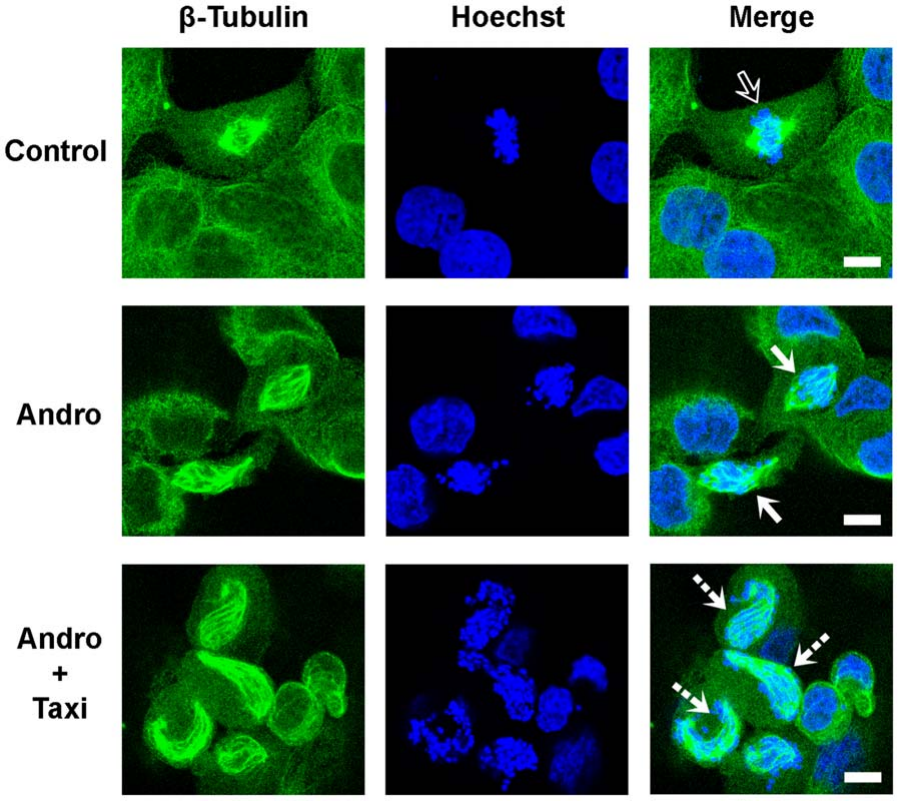
Immunofluorescence microscopic images of treating DU145 cells with Andro and Taxi on the spindle-chromosome configuration. The cells were treated with 20 µM of Andro in the presence and absence of 100 µM of Taxi for 24 h. The treated cells were fixed and immunostained with anti-tubulin antibody and FITC conjugated secondary antibody to visualize microtubules (green) and Hoechst 33342 to visualize chromosomes (blue). Representative images of spindle organization in the cells treated with vehicle control (hollow arrow), Andro alone (solid arrows) and combined Andro and Taxi (dashed arrows) are shown. Bar = 10 µm.

The increase in the mitotic index and rounding of cells treated with the two drugs suggested that these changes in the cells may be caused by the effects of the two drugs on microtubule dynamics or spindle assembly. Therefore, we investigated whether microtubule-based spindle formation was affected using fluorescence microscopy. When treated with DMSO or 100 µM Taxi alone, most of the cells were in interphase. Approximately 3% of the cells were in mitosis with a proper spindle-chromosome configuration at various stages, including metaphase (control, top row of Figure 9), where chromosomes align at the equator of the cells (indicated by the hollow arrow). After treatment with 20 µM Andro, the occurrence of mitotic cells increased considerably. However, cells in late metaphase, anaphase or telophase were rarely found. Instead, most of the chromosomes were condensed as dots and were scattered within the spindles, which were distorted in shape in some of the treated cells (solid arrows, second row). In most mitotic cells, the astral microtubules disappeared. This observation suggested that Andro alone at this concentration (20 µM) may disturb the proper formation of mitotic spindles in the cells. We have shown that Andro alone can induce DNA damage by altering the redox states in cells [16], [31]. The formation of small dot-shaped chromosomes may be due to limited DNA damage induced by Andro, which is consistent with the finding that during apoptosis, DU145 cells do not form DNA ladders typical of other apoptotic cells but instead form DNA fragments that are 50 kb long (51).

Of the cells treated with both drugs, a substantial proportion of the cells in mitotic arrest were found to exhibit spindles with aberrant structures to which the dot-like condensed chromosomes failed to align. However, the spindles in these cells were twisted and elongated (dashed arrows, third row, Figure 9). Comparison between the lengths of the microtubules along the spindles in these cells with microtubule lengths in the normal cells suggests that the microtubules of the spindles may be extended longer in the cells treated with both Andro and Taxi compared with the control.

**Figure 10:**
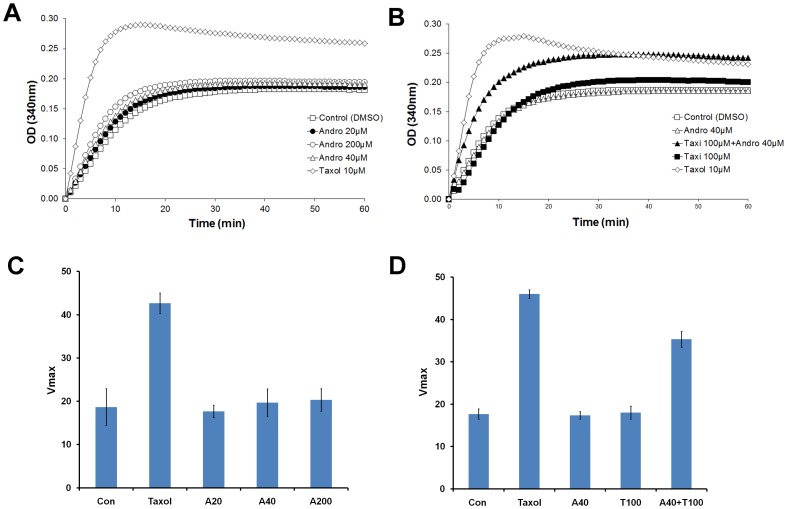
The combined effect of Andro and Taxi on enhancing tubulin assembly *in vitro*. Tubulin was incubated at 37°C in the reaction buffer in the presence of (A) 1% DMSO (vehicle control), Andro (20, 40 or 200 µM) or 10 µM of Taxol (positive control); (B) 1% DMSO; 100 µM Taxi; 40 µM Andro; 40 µM Andro+100 µM Taxi; or 10 µM Taxol. Tubulin polymerization was monitored by measuring the optical density at 340 nm for 60 min. Data are representative of three independent experiments. (C and D) The rate of increase in OD, Vmax, of the indicated conditions was plotted. The data represent the mean ± s.d. from three independent experiments.

To test whether microtubule dynamics were directly affected by the two drugs *in vitro*, a tubulin polymerization assay was used to determine how the two drugs affected the polymerization of purified tubulin molecules. Taxol, a microtubule stabilizing agent, was used at 10 µM as a reference compound for the assay. Taxol increased V*_max_* 2.6-fold (Figures 10C and D), and the final polymerized mass was much higher compared to the other controls (Figure 10A). Andro did not exert a significant effect on microtubule dynamics compared to vehicle control even up to a very high concentration (200 µM) (Figures 10A and C). However, when Andro (40 µM) was combined with Taxi (100 µM), V*_max_*, the initial rate of polymerization, increased to twice the rate of the control (n = 3). These results suggest that Taxi may modify microtubule stability and/or tubulin polymerization only when Andro is present.

**Figure 11:**
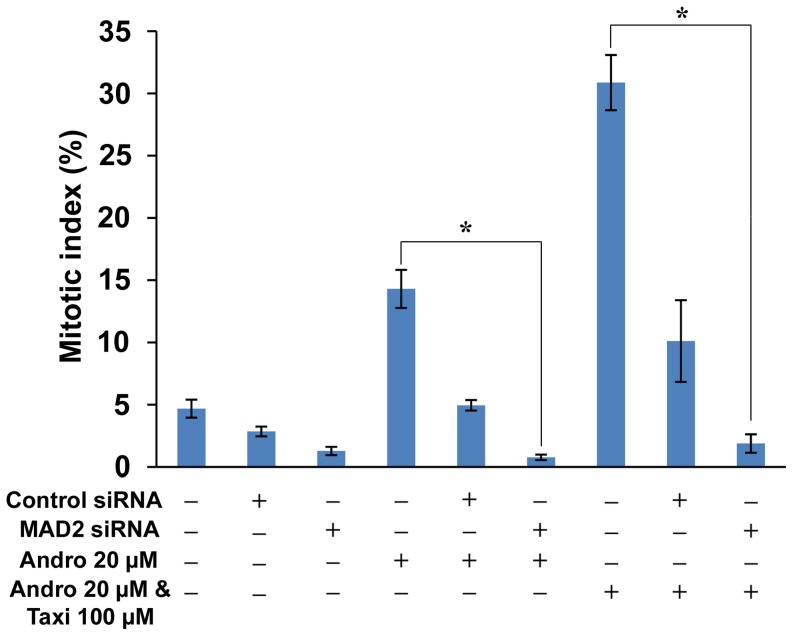
The effect of MAD2 knockdown on the mitotic index of DU145 cells treated with Andro and/or Taxi. Cells were either treated with Andro and/or Taxi for 24 h or transfected with the negative control siRNA or MAD2 specific siRNA for 24 h and later treated with Andro and/or Taxi for additional 24 h. Then cells were fixed and stained with anti-phospho-histone H3 (Ser10) antibody, FITC-conjugated secondary antibody and PI, and analyzed by flow cytometry. Mitotic index is expressed as mean ± s.d. * indicates significant difference between two groups, *p<0.01.

Deduced from the above experiments, we concluded that Andro itself cannot affect microtubule polymerization but that it can interfere with spindle assembly. With the addition of Taxi, they act together to enhance microtubule polymerization or stabilization *in vitro* and induce the formation of a long, twisted spindle. Therefore, we hypothesize that the malformation of the spindle induced by the two drugs may trigger the SAC. To demonstrate that the SAC is involved in mitotic arrest caused by Andro and Taxi, we transfected DU145 cells with two different siRNAs against MAD2 individually, a major protein involved in the SAC, before treating the cells with Andro and/or Taxi and then determining the mitotic index by flow cytometry (Figure S3). To show that the MAD2 siRNA was effective in depleting MAD2 protein, Western blotting was performed on the proteins from the cells treated with the MAD2 siRNA and showed that an average of 72% of the MAD2 protein was depleted from the cells (Figure S4). Flow cytometry analysis showed that 10.11±3.28% of the cell population was arrested in mitosis when the control double-stranded RNA-transfected cells (as a negative control) were treated with the two drugs, while the average percentage of mitotic arrested cells transfected with one of the two siRNAs against MAD2 decreased to 1.88±0.74% after the two-drug treatment (Figure 11). Quite unexpectedly, we found that the transfection reagent itself on the treated cells could lower the mitotic population (comparing Figures S3H with S3G), but the rationale behind this decrease is not clear. In addition, we found that Andro alone also activated the SAC as the mitotic cell population decreased from 4.95%±0.43 to 0.77%±0.22 when the cells were transfected with control siRNA vs. MAD2-specific siRNA, respectively (Figures 11). This result suggests that either Andro alone or the two drugs together can trigger the SAC, resulting in mitotic arrest of treated cells.

## Discussion

### Andrographolide halts the cell cycle of DU145 cells at prometaphase

Androgen-insensitive DU145 cells derived from human prostate adenocarcinoma at the metastatic stage are a commonly used cell model for advanced metastatic prostate cancer, which currently has no effective cure. An ideal therapeutic approach is to develop drugs that selectively target tumors while sparing normal tissues. Phytochemicals and their derivatives extracted from functional herbs may be a huge source for those agents. In this study, we found that Andro alone induced the accumulation of mitotic cells accompanied by a decrease in G0/G1 cells (Figure 2). This observation is consistent with the results of another related study from our group [16], but it differs from previous studies reporting that the inhibitory effect of Andro on cancer cell proliferation is due to cell cycle arrest in the G0/G1 phase [13]. Our examination regarding spindle organization showed that Andro treatment blocked the cell cycle at prometaphase (Figure 9), suggesting that it may have an effect on spindle formation or microtubule dynamics *in vivo*. Western blot analysis of cell cycle proteins showed that the Andro-treated cells accumulated cyclin B1, hyper-phosphorylated Myt1, and phosphorylated p21 proteins, indicating that mitotic cells accumulated with drug treatment.

However, in the present study, assessments of apoptosis induced in DU145 cells demonstrated that Andro was not a very efficient inducer of apoptosis within the concentration range examined. Although the compound triggered apoptosis in a concentration-dependent manner (Figure 3), the apoptotic rate was much lower than the mitotic accumulation rate when treated with the same concentration of Andro. This result may have been due to much higher concentrations of Andro being required for the inhibition of DNA synthesis, which leads to apoptotic progression; however, the inhibition of cell proliferation and cell arrest in certain cancer cells can be affected by lower concentrations of Andro [32].

### Andrographolide induces apoptosis via the intrinsic apoptotic pathway

PARP, a classical caspase substrate, is involved in DNA repair in response to environmental stress and in the maintenance of cell viability. Cleavage of PARP is regarded as a hallmark of apoptosis [33]. PARP cleavage proceeds simultaneously with cleavage of procaspase 3, 7, and 9 in a concentration-dependent manner (Figure 5A), indicating that Andro-induced apoptosis in DU145 cells is mediated through an intrinsic apoptosis pathway. Bcl-2 family members are the major regulators of apoptosis; therefore, the expression levels of these proteins in the two different subfamilies were assessed in our experiments. In the anti-apoptotic subfamily, Bcl-2 was only weakly expressed in both the treated and control cells, but Bcl-xL expression was clearly detected. In contrast, the expression of Bax, a pro-apoptotic protein, could not be detected (Figure 5B). These results are consistent with other previous studies showing that DU145 cells carry a loss-of-function mutation in p53, a mono-allelic Bax frameshift mutation, and a second silent Bax allele. Consequently, the expression of the proapoptotic protein Bax is completely lost in the cell [34]–[36]. Moreover, DU145 cells express a high level of Bcl-xL but lack Bcl-2 expression. Thus, Bcl-xL plays a major role as an anti-apoptotic regulator. Phosphorylated Bcl-xL is very likely to participate in the cell death process, as previous studies demonstrate that the phosphorylation of Bcl-2 and Bcl-xL may impair their anti-apoptotic functions and contribute to apoptosis [37]. The treated cells undergo apoptosis 48 h after a prolonged blockage in M phase. The low rate of apoptotic cell death in response to Andro is most likely due to the loss of Bax protein expression as proposed by Wendt *et al.* [38].

### Taxifolin enhances the anti-proliferation and apoptotic effects of Andrographolide

Many naturally occurring compounds have been suggested to act as “enhancers” or to reduce the side effects of other anti-cancer therapeutic drugs by increasing the therapeutic effects of the key anti-cancer agent [1], [2]. In this study, we showed that Taxi, a low cytotoxicity dietary flavonoid, markedly augments the anti-proliferation activity of Andro (Figure 6). We also found that the enhancement of growth inhibition in DU145 cells was likely due to potentiated mitotic accumulation (Figure 7) and subsequent apoptosis (Figure 8). Enhancements in the anti-proliferative effect, rate of mitotic blockade, and apoptotic rate at 20 µM Andro and 100 µM Taxi doubled when compared to cells treated with 20 µM Andro alone. However, a single exposure to 100 µM Taxi had no significant effect on cell proliferation or viability (Figure 1B and 6). Therefore, Taxi may act as an “enhancer” or “supporter” in Andro-induced cell-cycle arrest and cell death of DU145 cells.

### Taxifolin and andrographolide exert their effects on the microtubule dynamics and the activation of the SAC

Microtubule-targeted naturally occurring compounds are potential chemotherapeutic drugs for many types of tumors. Different anti-microtubule agents, such as paclitaxel, vinblastine, and nocodazole, exert their effects on tubulin polymerization through different mechanisms, but they commonly induce cell-cycle arrest in mitosis and subsequent apoptosis [39]. Andro exhibits typical features of anti-microtubule agents, such as mitotic arrest, activation of the cyclin B/Cdc2 complex and phosphorylation of Bcl-xL, which are the characteristic hallmarks of mitotic arrest induced by anti-microtubule agents [40], [41]. However, the results of the *in vitro* tubulin polymerization assay argue that at low concentration Andro has no direct effect on microtubule polymerization (Figure 10A). With the same concentration of Andro, the treated DU145 cells exhibited distorted spindles that were less frequent and less exaggerated than the cells treated with Andro and Taxi. We postulate that Andro may have an inhibitory effect on the destabilization activity of certain kinesin motor proteins necessary for spindle formation during mitosis.

The *in vitro* microtubule polymerization assay also showed that only when Andro and Taxi are added together in the reaction mixture, microtubule polymerization (microtubule-stabilizing effect) is promoted, increasing both the rate of tubulin polymerization and the final polymerized mass of the microtubule (Figures 10B and D). Although the exact nature of the synergistic effect of Andro and Taxi is not clear, we propose that the interaction between tubulin molecules and either chemical alone is too weak in the tubulin polymerization assay but it becomes much stronger when both chemicals are present due to the increase in the number of possible hydrogen bonds. The presence of these hydroxyl groups in both Taxi and Andro may help the interaction between tubulin monomers to form polymers. Also, a study has shown that Taxi can promote the stabilization of purified fibrillar collagen by its hydrophobic aromatic rings and forms hydrogen bonds between protein fibers [42].

Unlike other microtubule interfering chemicals, the combination of Andro and Taxi induces the formation of a more aberrantly twisted and elongated spindle structure in mitotic DU145 cells (Figure 9). Interestingly, Goshima and Vale found a similar observation regarding the formation of twisted and elongated mitotic spindles after knockdown of Klp67A alone (a microtubule destabilizing kinesin) or both Klp67A and BubR1 (a spindle checkpoint protein) in *Drosophila* S2 cells, suggesting disrupting the proteins involved in spindle formation and disintegration may lead to the formation of twisted and elongated spindle fiber [43]. It is likely that in DU145 cells, the inhibitory effect of Andro on certain kinesin motor proteins and the tubulin polymerization effect of Andro and Taxi in combination further exaggerate the stabilization and curving of spindle fibers, leading to the subsequent activation of the SAC during mitosis. We also believe that besides the formation of twisted spindle, Andro can affect the redox balance in cells and causes oxidative damage to DNA, which in turn may lead to limited DNA cleavage and chromosome fragmentation in DU145 cells [44].

From the MAD2 depletion experiment using siRNA, the accumulation of mitotic cells after the addition of either Andro alone or Andro and Taxi is caused by the activation of the spindle assembly checkpoint. The stalling of cell cycle progression in mitosis by the SAC in the treated cells can account for the increase in the levels of cyclin B, Cdc25c and p21 proteins.

In conclusion, the present study demonstrates that Andro induces mitotic arrest at G2/M and the activation of the apoptotic pathway. Furthermore, the flavonoid Taxi strongly potentiates Andro-induced mitotic arrest and apoptosis due to its enhanced microtubule-stabilizing effect, which disturbs the normal function of mitotic spindles, leading to further mitotic arrest and the activation of apoptotic signaling cascades. This study provides experimental evidence that Taxi and possibly other flavonoids with a similar molecular structure may act as “enhancers” in combination with Andro to treat prostate cancer.

## Supporting Information

Figure S1 **Cellular morphological changes of DU145 cells treated with Andro.** The cells were treated with Andro at different concentrations for different time periods and then observed with phase contrast microscopy. (A) control, 48 h; (B) 80 µM Andro, 24 h; (C) 20 µM Andro, 48 h; (D) 20 µM Andro, 72 h; (E) 40 µM Andro, 48 h; (F) 40 µM Andro, 72 h. Blue arrows indicate round and loosely-attached cell and pink arrows indicated apoptotic body formation. Bar = 50 µm.
(TIF)

Figure S2 **Nuclear morphological change of DU145 cells treated with Andro.** The cells were treated with Andro at different concentration for 48 or 72 hours; adherent cells and floating cells were stained by Hoechst 33342 and observed under fluorescence microscopy respectively. A) control, 48 h; (B) 20 µM Andro, 48 h, adherent cells; (C) 20 µM Andro, 72 h, adherent cells; (D) 20 µM Andro, 48 h, adherent cells; (E) 40 µM Andro, 72 h, adherent cells; (F, G) 40 µM Andro, 48 h, floating cells. Yellow arrows indicate nuclear condensation and red arrows indicate nuclear fragmentation. Bar = 50 µm.
(TIF)

Figure S3 **MAD2 depletion abrogates Taxi+Andro-induced mitotic block.** DU145 cells were transfected with or without a MAD2-specific double-strand siRNA and then incubated further for 24 h in DMEM before treatment with 20 µM Andro alone or 20 µM Andro and 100 µM Taxifolin or without any chemical for 24 h. After fixation, the cells were analyzed with flow cytometry for the population (in percentage) of mitotic cells (in boxes) with higher levels of phospho-histone H3 (at S10) and propidium iodide signals. The cells were: (A) both non-transfected and non-treated with the two drugs, (B) transfected with a control siRNA and non-treated with the two drugs, (C) transfected with a MAD2 specific siRNA and non-treated with the two drugs, (D) not transfected but treated with Andro, (E) transfected with control siRNA and treated with Andro, (F) transfected with a MAD2 specific siRNA and treated with Andro, (G) not transfected but treated with Andro and Taxi, (H) transfected with a control siRNA and treated with Andro and Taxi and (I) transfected with a MAD2 specific siRNA and treated with Andro and Taxi. A representative dot plot from three independent experiments for each treatment is shown here. The numbers in the box are the mean and standard deviation of the percentage of mitotic cells from three independent experiments except in (H) the number refers to the average of the mitotic population from two individual MAD2-specific siRNAs, with each performed in triplicate.
(TIF)

Figure S4 **Double-strand siRNA depletion of MAD2 protein in DU145 cells.** DU145 cells were transfected with three different siRNA against MAD2 gene or control siRNA for 24 h and the protein extracts from these cells were separated and analyzed with an anti-MAD2 specific antibody (upper panel) and actin antibody (lower panel). The actin control was used for normalization of loading of protein in each lane.
(TIF)
