# Supplementary figures and images for: Taxifolin Enhances Andrographolide-Induced Mitotic Arrest and Apoptosis in Human Prostate Cancer Cells via Spindle Assembly Checkpoint Activation
Source: PLoS One. 2013 Jan 28;8(1):e54577. doi: 10.1371/journal.pone.0054577 (PMC3557238; doi:10.1371/journal.pone.0054577)

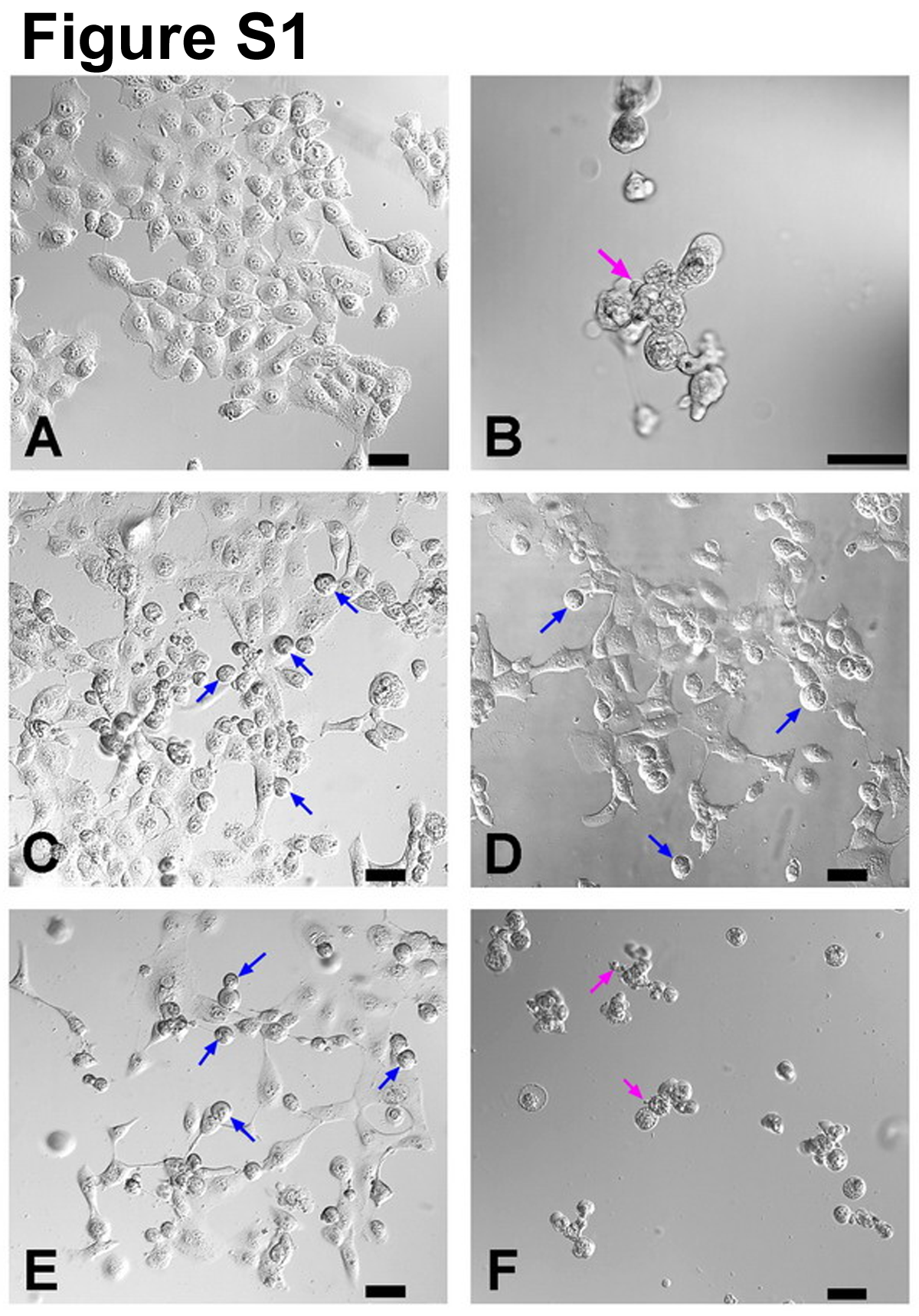

Supplement: Figure S1 — Cellular morphological changes of DU145 cells treated with Andro. The cells were treated with Andro at different concentrations for different time periods and then observed with phase contrast microscopy. (A) control, 48 h; (B) 80 µM Andro, 24 h; (C) 20 µM Andro, 48 h; (D) 20 µM Andro, 72 h; (E) 40 µM Andro, 48 h; (F) 40 µM Andro, 72 h. Blue arrows indicate round and loosely-attached cell and pink arrows indicated apoptotic body formation. Bar = 50 µm. (TIF) [file pone.0054577.s001.tif]

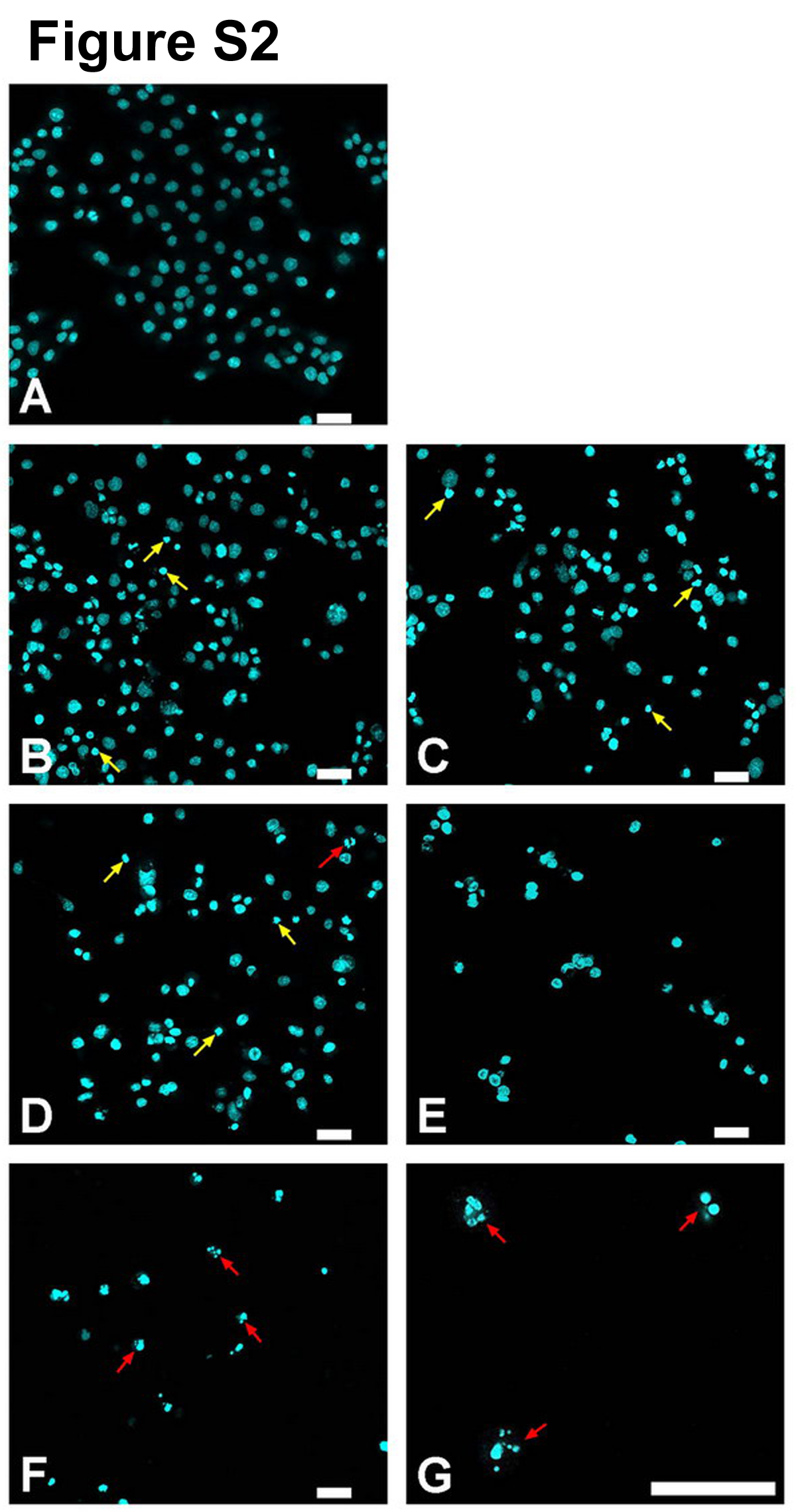

Supplement: Figure S2 — Nuclear morphological change of DU145 cells treated with Andro. The cells were treated with Andro at different concentration for 48 or 72 hours; adherent cells and floating cells were stained by Hoechst 33342 and observed under fluorescence microscopy respectively. A) control, 48 h; (B) 20 µM Andro, 48 h, adherent cells; (C) 20 µM Andro, 72 h, adherent cells; (D) 20 µM Andro, 48 h, adherent cells; (E) 40 µM Andro, 72 h, adherent cells; (F, G) 40 µM Andro, 48 h, floating cells. Yellow arrows indicate nuclear condensation and red arrows indicate nuclear fragmentation. Bar = 50 µm. (TIF) [file pone.0054577.s002.tif]

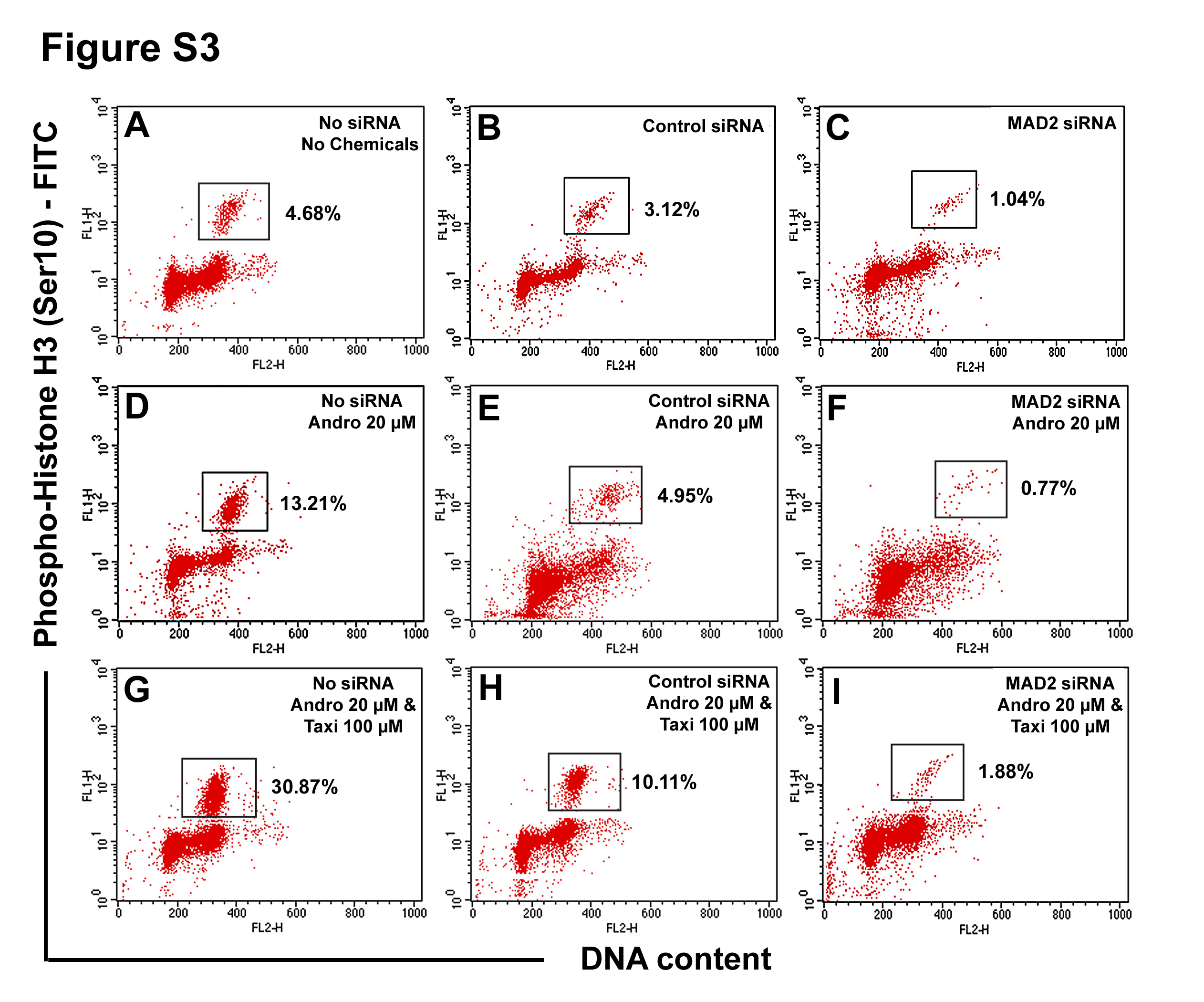

Supplement: Figure S3 — MAD2 depletion abrogates Taxi+Andro-induced mitotic block. DU145 cells were transfected with or without a MAD2-specific double-strand siRNA and then incubated further for 24 h in DMEM before treatment with 20 µM Andro alone or 20 µM Andro and 100 µM Taxifolin or without any chemical for 24 h. After fixation, the cells were analyzed with flow cytometry for the population (in percentage) of mitotic cells (in boxes) with higher levels of phospho-histone H3 (at S10) and propidium iodide signals. The cells were: (A) both non-transfected and non-treated with the two drugs, (B) transfected with a control siRNA and non-treated with the two drugs, (C) transfected with a MAD2 specific siRNA and non-treated with the two drugs, (D) not transfected but treated with Andro, (E) transfected with control siRNA and treated with Andro, (F) transfected with a MAD2 specific siRNA and treated with Andro, (G) not transfected but treated with Andro and Taxi, (H) transfected with a control siRNA and treated with Andro and Taxi and (I) transfected with a MAD2 specific siRNA and treated with Andro and Taxi. A representative dot plot from three independent experiments for each treatment is shown here. The numbers in the box are the mean and standard deviation of the percentage of mitotic cells from three independent experiments except in (H) the number refers to the average of the mitotic population from two individual MAD2-specific siRNAs, with each performed in triplicate. (TIF) [file pone.0054577.s003.tif]

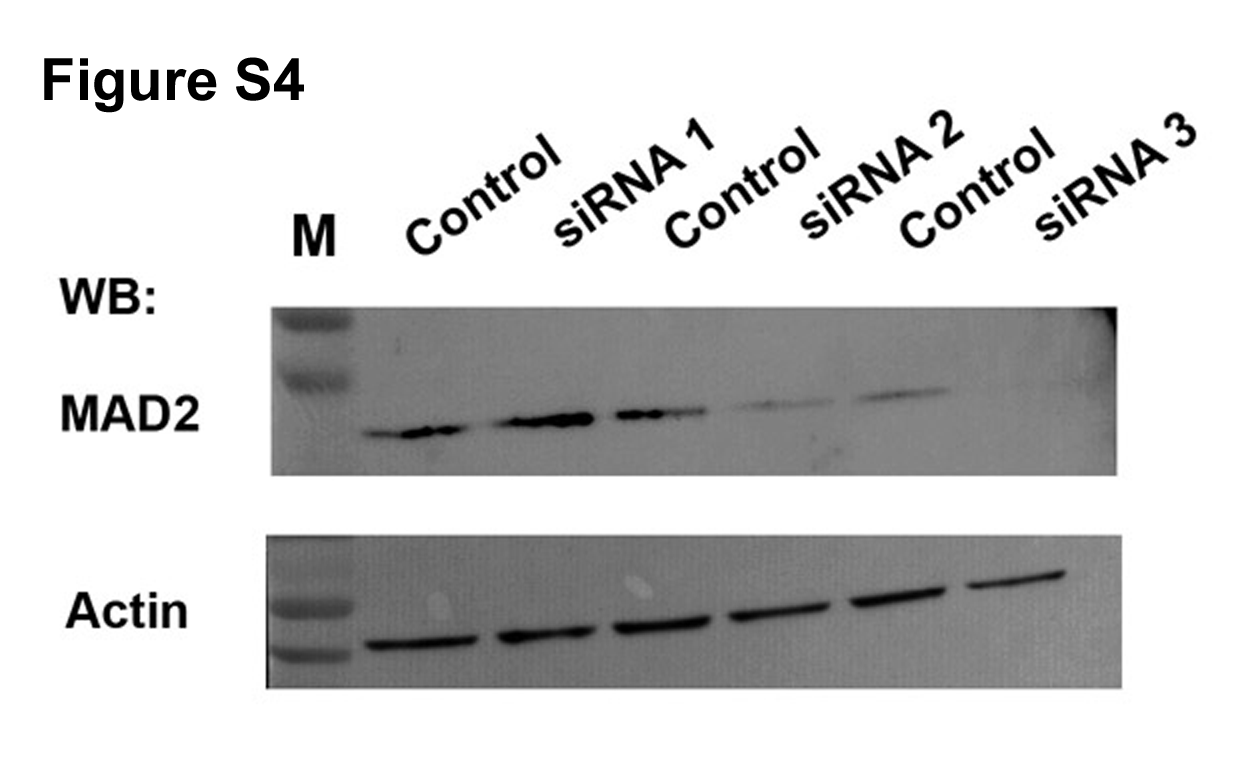

Supplement: Figure S4 — Double-strand siRNA depletion of MAD2 protein in DU145 cells. DU145 cells were transfected with three different siRNA against MAD2 gene or control siRNA for 24 h and the protein extracts from these cells were separated and analyzed with an anti-MAD2 specific antibody (upper panel) and actin antibody (lower panel). The actin control was used for normalization of loading of protein in each lane. (TIF) [file pone.0054577.s004.tif]
